# Digital twins to accelerate target identification and drug development for immune‐mediated disorders

**DOI:** 10.1002/2211-5463.70228

**Published:** 2026-03-23

**Authors:** Anna Niarakis, Philippe Moingeon

**Affiliations:** ^1^ Computational System Biology Team, Laboratory of Molecular, Cellular and Developmental Biology, Centre for Integrative Biology, CNRS Toulouse University Toulouse France; ^2^ UFR Pharmacy Paris Saclay University Orsay France

**Keywords:** artificial intelligence, computational biology, digital twin, drug development, precision medicine, virtual patient

## Abstract

Immune‐related/mediated disorders (IDs) comprise a very diverse group of diseases affecting millions worldwide. The complexity and heterogeneity of IDs, coupled with individual variability in immune system responses, create multiple challenges for developing targeted therapies. These challenges often result in prolonged diagnostic timelines, higher treatment costs, and frequent failures in clinical trials. Recent advances in artificial intelligence (AI) and digital twin (DT) technology offer promising solutions to support and accelerate drug discovery and development for these conditions, with anticipated substantial improvement in success rates. As virtual replicas of biological systems, DTs can be constructed using multimodal data sources, including multi‐omics, molecular profiling, imaging and clinical records. These *in silico* tools can accelerate precision medicine by identifying relevant drug targets, designing personalised treatments and predicting individual immune responses to drug candidates. Here, we review the current landscape of DTs supporting drug development for IDs. We describe the concepts behind mixed reality approaches combining AI‐based models, traditional mathematical and computational models based on low‐throughput experiments and empirical studies. We highlight concrete examples of precision medicine strategies for IDs informed by computational modelling. We also address the benefits, limitations, and ethical considerations of these approaches, and outline future directions for research and clinical translation.

**Impact statement**

This manuscript addresses the use of digital twins (DT) to accelerate drug discovery and development for Immune‐mediated Disorders. It provides a comprehensive overview of the field and helps clarify complex concepts. Furthermore, it provides concrete examples of DT applications on immune‐mediated disorders, and discusses perspectives, and current challenges.

## Graphical abstract

Digital twins integrate patient‐derived molecular and clinical data into personalised computational models that simulate disease mechanisms. They enable rapid identification and validation of therapeutic targets, prediction of drug responses, and prioritisation of candidate interventions. By linking biology with *in silico* testing, digital twins can accelerate drug development and support more precise treatments for immune‐mediated disorders. Graphical Abstract created using https://biographics.nitro.bio/.

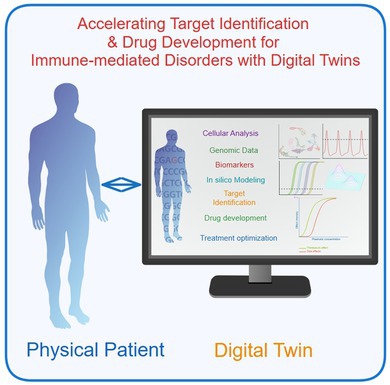

**Abbreviations**
ADMET: absorption, distribution, metabolism, excretion, toxicity
AI: artificial intelligence
DL: deep learning
DT: digital twin
ID: immune disorder
ML: machine learning
QSAR: quantitative structure activity relationship
QSP: quantitative systems pharmacology
VP: virtual patient
XAI: explainable AI

Immune disorders (IDs) encompass a broad spectrum of conditions characterised by altered immune responses, either as a cause or a consequence of the disease. Such conditions include autoimmune diseases, allergies, cancers, primary immunodeficiencies, infectious and autoinflammatory diseases [1, 2, 3, 4, 5, 6, 7, 8]. Autoimmune diseases, including rheumatoid arthritis, type 1 diabetes, Lupus erythematosus and multiple sclerosis, arise when the immune system mistakenly attacks the body's own tissues [4]. Allergies, ranging from mild seasonal reactions to life‐threatening anaphylaxis, result from inappropriate immune responses to otherwise harmless substances [6]. Immunodeficiencies, whether inherited or acquired, as well as hematologic malignancies such as leukaemias and lymphoma, leave individuals susceptible to frequent and severe infections [8]. Infectious diseases can be associated with severe immune impairment, as illustrated by the recent COVID‐19 crisis [2, 9, 10]. Lastly, autoinflammatory diseases involve the hyperactivation of immune pathways leading to pathogenic inflammation in an antigen‐independent fashion [11, 12]. Collectively, these acute or chronic disorders represent a significant and growing burden on global health, affecting hundreds of millions of people worldwide, resulting in significant morbidity, reduced quality of life, and substantial healthcare costs.

Despite advances in biomedical research, developing targeted therapies for IDs remains challenging. The complexity of the immune system, involving a vast network of cells, signalling molecules, and feedback mechanisms, in combination with the individual variability in physiological or altered immune responses driven by genetics, environment and lifestyle, complicates the prediction of both natural disease progression and therapeutic responses [4, 5]. Consequently, traditional drug development approaches are lengthy and expensive, with high rates of attrition due to unforeseen side effects or lack of efficacy. The documented heterogeneity of patients with IDs means that a “one‐size‐fits‐all” approach is often ineffective, highlighting the need for more personalised treatment strategies. In this context, an evolution towards precision medicine approaches is being proposed to tackle various IDs, with innovative drugs targeted to patient subsets with defined molecular profiles [13, 14].

Advances in artificial intelligence (AI) and data‐driven statistical and computational approaches, such as machine learning (ML) and deep learning (DL), are poised to revolutionize the landscape of drug discovery and development [15, 16, 17] by enabling new capacities for data integration and predictive modelling. AI encompasses a range of computational techniques that enable machines to perform tasks typically requiring human intelligence [15]. In healthcare, ML algorithms can uncover patterns in large, complex datasets, while DL—using neural networks with multiple layers—excels at processing data such as medical images or multi‐omics profiling [16]. Predictive modelling leverages these techniques to forecast disease risk, treatment outcomes, or adverse events. Powerful tools such as digital twins (DTs), first introduced by the aerospace and automotive industries to create virtual models of physical objects, are now used to produce virtual representations of complex biological entities, such as the human immune system, leveraging data and prior knowledge [18, 19, 20, 21]. DTs can be designed to create highly detailed, personalised models that can predict disease progression, identify novel therapeutic targets, simulate drug responses, and optimise treatment regimens before clinical trials have been initiated [18, 19].

In this context, we explore the use of DT technologies as a transformative approach to accelerate targeted drug development to support precision medicine with an application to immune‐related pathologies. We first outline specific challenges arising from the complexity of the immune system, along with the new functionalities enabled by DTs to address them. We then examine concrete examples from recent research and clinical applications focused on developing new treatments for IDs. Finally, we discuss the benefits, limitations, and ethical considerations associated with DT model implementation in the clinic, and present future perspectives for its adoption to envision safer, more effective therapies better tailored to the unique needs of individuals living with IDs.

## Towards precision medicine for immune disorders

### Facing the challenge of biological complexity

The immune system is a dynamic and intricate network that defends the body against external pathogens while maintaining self‐tolerance. It comprises innate and adaptive branches, each involving diverse cell types, including macrophages, dendritic cells, T cells, and B cells. These effector cells communicate through a sophisticated array of signalling molecules, including cytokines and chemokines, orchestrating rapid, precise and memory‐sustained immune responses. A significant challenge in various IDs, where the delicate balance of self‐tolerance is disrupted, lies in the heterogeneity and profound individual variability in disease manifestation and progression [1, 4]. Genetic differences, environmental exposures, microbiome composition, and lifestyle choices all contribute to the highly variable immune responses. Consequently, patients with the same diagnosis may respond quite differently to standard treatments. This heterogeneity complicates the development of universally effective therapies and underscores the need for personalised approaches that account for each individual's unique immunological landscape and genetic makeup [5, 13].

### The rise of digital twins in health

The concept of a DT originated in the aerospace and automotive industries, where it refers to a virtual replica of a physical object, system, or process. These digital counterparts are continuously updated with real‐world data, enabling simulation, monitoring, and optimisation throughout the asset's lifecycle [19, 21]. In healthcare, digital twins are emerging as powerful tools for modelling complex biological systems, including the health status of individual patients. These DTs integrate diverse data—such as expression data (genomics, transcriptomics, epigenetics, proteomics, metabolomics, deep immunophenotyping), high‐resolution imaging data, clinical history, and wearable sensor outputs—allowing the construction of a personalised, dynamic model of an organ, or to represent individual patients' disease status [18, 19, 20, 21, 22]. The latter could then be used to simulate disease progression, predict responses to therapeutic interventions, and inform clinical decision‐making, offering the most appropriate, safe, and effective treatment for a given patient.

Relevant DTs in biomedical sciences are emerging, primarily thanks to AI's capacity to integrate and analyse vast, heterogeneous datasets to build models supporting drug discovery [18, 19, 20, 21, 22]. AI, and particularly machine learning, plays a critical role in refining DTs, as learning algorithms can identify patterns and relationships within vast, complex datasets that may not be apparent to human experts [18, 19, 20, 21, 22]. These insights can be used to calibrate model parameters, optimise simulation accuracy, and identify sources of error or uncertainty in traditional modelling approaches.

AI is increasingly being integrated into the development of large‐scale computational models and DTs, complementing more traditional modelling approaches such as ordinary differential equation (ODE) models, rule‐based systems, agent‐based simulations and logic‐based formalisms [23, 24, 25]. Traditional models provide mechanistic insights into biological and clinical systems, capturing causal relationships and system dynamics based on known processes. However, they often require detailed prior knowledge and can struggle with high‐dimensional, noisy data [26, 27]. AI methods—ranging from ML to DL—offer powerful tools for pattern recognition, data integration, and predictive analytics, enabling digital twins to assimilate diverse data streams (omics, imaging, clinical records) and adapt in near real time. By linking data‐driven AI with mechanistic models, DTs can combine interpretability with predictive accuracy, creating a hybrid framework that is both biologically grounded and flexible enough to evolve with new evidence.

### Building digital twins of immune‐related disorders

As stated above, constructing a digital twin of the immune system begins with comprehensive data collection across multiple biological domains [19, 21, 28]. DNA sequencing reveals genetic variants that influence immune function, disease susceptibility, and drug metabolism. Transcriptomic and protein expression profiles provide insights into the dynamic state of immune cells and signalling pathways, together with a comprehensive phenotypic and functional characterisation of immune cell subsets based on the analysis of surface‐expressed and intracellular markers. Moreover, advances in single‐cell sequencing have revolutionized the way we analyse and identify molecular programs and cellular machineries that operate in a given cell type. Electronic health records, laboratory results, imaging, and patient‐reported outcomes offer a longitudinal view of disease progression and treatment response. Information on diet, microbiome composition, exposures, and lifestyle habits further contextualises individual immune variability [22].

**Fig. 1:**
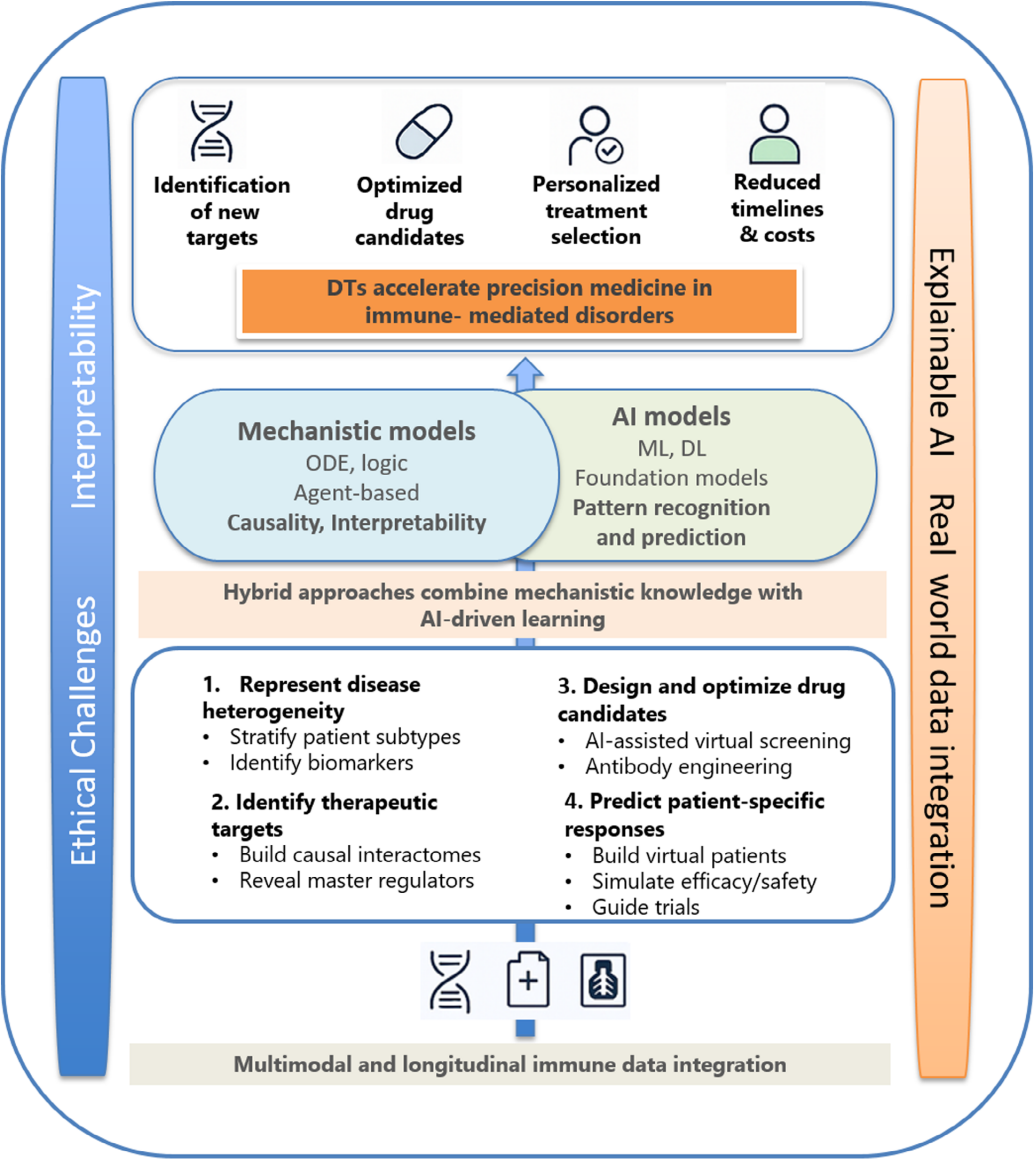
Schematic representation of bioinformatics pipelines that implement computational modelling (mechanistic or Artificial Intelligence/Machine Learning driven) to develop Digital Twins for target identification and drug development for immune‐mediated disorders. AI, artificial intelligence; DL, deep learning; DT, digital twins; ML, machine learning; ODE, ordinary differential equation.

The integration of these massive and multimodal data sources requires appropriate data management systems and standardised formats to ensure interoperability and consistency. It should allow multi‐scale modelling, essential to reflect the dynamics of the immune system operating across scales, spanning molecular interactions within cells to complex feedback loops at the tissue and systemic levels [19, 21, 28] (Fig. 1). DTs must therefore simulate, at the cellular level, intracellular signalling, gene regulation, and cell differentiation. At a tissue level, interactions between immune cells, stromal cells, and pathogens should be captured within specific microenvironments. At a systemic level, the circulation of immune cells and molecules, organ‐to‐organ communication, and whole‐body responses should be represented [19, 21, 28]. Moreover, specific characteristics, such as age and biological sex, should be taken into account, particularly when the DT is used to identify or optimise a treatment scheme.

## Applications of digital twins to support drug development for immune disorders

The focus of this section is to identify applications of digital twin technology to support drug development for IDs, regardless of the type of DT used (AI–enabled, ML–based, hybrid, or mechanistic). While a DT, by strict definition, is a two‐way information system connected to the physical object and capable of continuous, real‐time updates, this definition is difficult to apply in biomedicine. In this review, we consider DTs' large‐scale computational models that use multimodal data to predict a patient‐related outcome. Schematically, four categories of applications are currently explored (Table 1). The latter include (a) DT models to understand and represent patient heterogeneity; (b) DTs to identify relevant therapeutic targets among dysregulated genes or proteins associated with the disease; (c) DTs modelling the interactions between the target and potential drugs, allowing selection of the appropriate therapeutic modality and further identification and optimisation of drug candidates with desired properties; (d) DTs used for *in silico* simulation of therapeutic scenarios and prediction of patient‐specific responses to drug candidates.

**Table 1:** Selected applications of digital twin models to immune‐mediated disorders.

DT model objectives	Case studies related to immune disorders	References
Representation of disease heterogeneity	DT models integrating genomic, transcriptomic, proteomic, metabolomic, and deep immunophenotyping data have been assembled to stratify patients with distinct molecular patterns in autoimmune diseases, such as rheumatoid arthritis, systemic Lupus erythematosus, primary Sjögren's syndrome, scleroderma, sepsis, inflammatory bowel disease (IBD), Crohn's disease and psoriasis. DT models are being used to identify biomarkers to stratify patients with those diseases	[33, 43, 44, 47, 64]
	Generative AI based on large language models can help categorise patients by using text mining of their medical records and contextual information. This approach was used to improve the diagnosis of hematologic malignancies and primary immunodeficiencies	[8, 14]
	DT models of severe forms of COVID‐19 (acute respiratory distress syndrome) have been created to identify and manage patients at risk	[10]
Identification of relevant therapeutic targets	DT models of various autoimmune diseases (Rheumatoid Arthritis, psoriasis, Systemic Lupus Erythematosus, multiple sclerosis) have confirmed dysregulation of numerous proinflammatory pathways, both disease‐specific and shared across multiple IDs	[23, 32, 35, 36, 37, 41, 42, 48, 65]
	Various DT models of COVID‐19 or sepsis have been generated to identify targets for drug repurposing or new treatments	[9, 10]
Selection and optimisation of therapeutic modalities and drug candidates	DT models are being used to optimise the design of therapeutic monoclonal antibodies, including binding to the target (based on nucleotide sequences from the variable regions of both heavy and light chains), as well as their developability (predicting manufacturing, immunogenicity, stability and the risk of forming aggregates)	[28, 38, 39]
	DTs can support the personalization of combination therapies. Simulations of interactions between multiple drugs and the identification of biomarkers have helped optimise therapeutic combinations for complex autoimmune diseases	[46, 47]
	DT models have been created to optimise CAR T‐cell therapy in myeloma and accelerate drug discovery for psoriasis	[49, 50]
*In silico* prediction of drug efficacy and safety	DT modelling of rheumatoid arthritis, integrating genomic, proteomic and clinical data, enabled the identification of subgroups likely to benefit from TNF‐α inhibitors versus those predicted to be non‐responders	[33, 51]
	A population of virtual patients has been created to simulate responses to a T cell‐engaging bispecific antibody in non‐Hodgkin lymphoma, accounting for different dosing regimens and patient heterogeneity	[52]
	Combining a mechanistic QSP model of cutaneous Lupus with ML, a virtual cohort of 20 000 virtual patients inspired by the molecular profiling of actual patients has been used to identify molecular signatures distinguishing predicted responders from non‐responders. This data can inform the design of confirmatory clinical studies, increasing chances of success and reducing patient exposure to ineffective treatments	[40, 41]
	In immuno‐oncology, DTs of the immune system have been used to simulate the efficacy of monoclonal antibodies and immune checkpoint inhibitors. AI models analyse tumour‐immune interactions and patient‐specific factors to forecast treatment outcomes and adverse events. These insights have guided the selection of combination therapies and dosing schedules, leading to more personalised and effective immunotherapy regimens	[52, 53]
	In Sepsis management, DT models of immune responses have been used to simulate the effects of various interventions, such as antibiotics or immune modulators. AI algorithms help predict which patients are at risk of rapid deterioration, enabling earlier and more targeted treatments	[54, 66]

### DT to understand and represent patient heterogeneity

A first major application of DT is to create disease models by leveraging omics profiling data from blood and target organs of large patient cohorts to dissect the complex molecular landscape of IDs and document patient heterogeneity [17, 19, 29]. Whereas the integration and analysis of such high‐dimensional data have previously been hindered by a lack of sophisticated computational methods, recent advancements in AI and ML have opened new opportunities to identify patterns in patient data, enabling the delineation of homogeneous patient subgroups and the identification of biomarkers indicative of disease subtypes. These advances, on the one hand, enhance understanding of disease pathophysiology, but also allow stratification of patients with a given ID into homogeneous subgroups based on molecular signatures [4, 21, 28]. This categorisation of patients, together with the identification of clinically relevant stratification biomarkers, is pivotal in the transition towards precision medicine, which accounts for patient‐specificities to adapt treatment modalities.

### DTs to identify therapeutic targets

DT models of disease play a crucial role in identifying relevant therapeutic targets from unique molecular patterns, such as dysregulated pathways in the blood or target organs. To further understand the mechanisms underlying complex IDs, computational modelling is being employed to determine which dysregulated genes or proteins are responsible for the disease. Recent advancements in AI, integrated with network biology approaches, enable the assessment of the impact of each disease‐associated gene or protein on the expression of all other components of the perturbed biological system [30]. Causal disease models are then created as interactomes of dysregulated genes or proteins that represent the pathophysiology [31]. Comparing these disease interactomes with those from healthy controls reveals significant differences in how master regulators influence the entire biological system [32, 33, 34]. Those genes or proteins that are likely to contribute to its causality are of interest as potential therapeutic targets. In addition, computationally perturbing the causal disease system, for instance by increasing or decreasing the level of influence of selected hubs in the network, allows simulating the effects of drugs targeting these specific targets [4, 30]. Logic‐based modelling and probabilistic simulations can compensate for the lack of data on specific diseases that could be used to train ML models [23, 27, 35, 36, 37].

### DTs to identify and select drug candidates

The identification of relevant targets likely involved in disease causality further instructs the design and optimisation of drug candidates to treat IDs. Candidates interacting with a therapeutic target of interest, typically a protein, can be selected using AI‐enhanced computational chemistry to predict high‐affinity binders among billions of small chemical molecules, based on quantitative structure–activity relationships (QSAR) [17]. The accurate prediction of 3D protein target structures from simple amino‐acid sequences with DeepMind's AlphaFold 3.0 algorithm facilitates the selection of high‐affinity binders based on structural complementarity [29, 38]. To further predict functional properties of small chemical molecules, ML training of convolutional neural networks on ligand‐based virtual screens enables the identification of drugs with desired properties across a vast chemical space of potential molecules. The optimisation of lead candidates is further performed by refining the decoration of these molecules and predicting their functional properties. As of today, current algorithms enable multitasking, parallel prediction of ADMET (absorption, distribution, metabolism, excretion, and toxicity) properties, as well as solubility and stability, all of which are critical for further drug development. Based on these predictions, only a limited number of drug candidates need to be synthesised for further evaluation in confirmatory laboratory tests, with a high success rate [17].

Whereas ML approaches based on neural networks have initially been applied to selecting drug candidates from small chemical molecules or synthetic antisense oligonucleotides, various DT models are now being used to design optimal therapeutic monoclonal antibodies, one of the preferred modalities for treating many IDs [21, 28, 39]. The computational models enable the prediction of high‐affinity binding to the target—as well as epitope recognition—from the nucleotide sequences of the variable regions of both heavy and light chains, thereby guiding the most appropriate pairing for antibody design. To support the developability of candidate monoclonal antibodies, additional algorithms can assess similarity to natural immunoglobulins to reduce immunogenicity, predict the risk of forming aggregates and assess manufacturability in expression systems [21, 24, 28, 39, 40, 41].

Lastly, large‐scale computational models can also be used to identify repurposed drugs for selected candidates [9, 36, 42].

### DTs to predict patient‐specific responses to drug candidates

DTs are used to predict *in silico* the performance of drug candidates, evaluating various dosing regimens, routes of administration, or combination therapies, before exposing patients to potential risks. Virtual patients (VPs) are a specific form of DT used to predict how an individual's immune system is likely to respond to each scenario, enabling the modelling of drug efficacy and safety [21, 29, 43]. VPs can be designed to represent individual patients, based on biological and clinical data from their medical records. Alternatively, synthetic patients can be generated from data compiled across multiple patients, thereby avoiding privacy concerns while providing a realistic representation of the disease. VPs can also be created from mechanistic quantitative systems pharmacology (QSP) models of a disease. The latter are established by integrating data documenting both disease‐related biological processes and the clinical symptoms of interest as endpoints, along with the pharmacokinetic and pharmacodynamic properties of the drug candidate [24, 40, 41]. Cohorts of thousands of VPs can thus be created by varying biological and clinical parameters in the QSP model, with AI and ML being used to identify signatures distinguishing VPs predicted to be responders from non‐responders to the drug candidate [24, 40, 41]. The identification of criteria and biomarkers to select real patients likely to benefit from the drug candidate is extremely valuable for designing confirmatory clinical studies, with a high chance of success.

As more real‐world data are incorporated, DTs become increasingly predictive and reliable. Selected concrete examples of each of the four outlined DT applications to ID management are presented in Table 1 [43, 44, 45, 46, 47, 48, 49, 50, 51, 52, 53, 54, 55]. These pioneering efforts have already demonstrated the feasibility and the benefits of integrating DT models powered by AI in immune‐related drug development.

## Benefits and challenges of digital twin models for immune disorders

### Confirmed benefits

Concrete DT applications have yielded several tangible benefits in ID management. DT models used in a mixed modality approach, that is, combined with high‐throughput empirical preclinical and clinical studies, considerably strengthen the scientific rationale—in terms of therapeutic target and drug candidate selection—to develop « intelligent drugs ». The latter refers to remarkably targeted drugs, both in the biological process they modulate and in the patient population they may benefit. Reduced costs and risks are enabled by narrowing candidate therapies before clinical testing, with better success rates anticipated in those costly studies. Aiming to personalise more ID treatments, DT simulations enable the selection of therapies better adapted for each patient, thus expected to improve efficacy and safety.

The introduction of DTs in support of ID management brings a series of additional transformative advantages. One of the most significant is the acceleration of drug discovery and validation processes. Traditionally, the development of new treatments for immune disorders has been a time‐consuming and resource‐intensive endeavour, requiring 5–8 years of laboratory work and extensive preclinical studies [56]. AI‐driven approaches can rapidly analyse vast datasets, identify promising targets for immune modulation, and predict the efficacy of potential drug candidates, thereby shortening the timeline from discovery to clinical testing. Continuous learning is achieved as more data are collected, thereby improving the accuracy of DT models.

Another anticipated key benefit is reduced costs from fewer clinical trials. By leveraging *in silico* models and AI‐based simulations, researchers can predict how patients might respond to specific therapies before conducting large‐scale clinical trials. This predictive power should enable prioritising the most promising candidates, reducing the number of ineffective or redundant trials. The design of confirmatory clinical trials in actual patients will benefit considerably from predictive outputs from DT models. Consequently, pharmaceutical companies and research institutions should eventually be able to allocate resources more efficiently, ultimately lowering the overall cost of bringing new therapies to market.

Furthermore, DT modelling will considerably strengthen our understanding of immune mechanisms. Computational models can uncover hidden patterns and relationships within high‐dimensional biological data, generating new hypotheses about immune function and dysfunction. For example, in support of system vaccinology, it can shed light on protective immune responses elicited by successful vaccines used against infectious pathogens or cancers [57]. This more profound understanding of immune processes could lead to breakthroughs in both prevention and treatment strategies. Lastly, personalised DTs could help elucidate autoimmune signatures, enriching existing biomarker candidates with patient‐specific targets. As the diagnosis of autoimmune pathologies is delayed and patients often start treatment after 4 years or more, accelerating the diagnosis can have a tremendous impact on patient response and delayed disease progression.

### Challenges and limitations

Despite the above benefits, several challenges and limitations must be addressed to fully realise the potential of computational modelling applied to IDs. A primary concern is model complexity and the need for high‐quality data. DT models, especially those based on AI and deep learning, require large, diverse and accurately labelled datasets to perform optimally. For some IDs, such data can be scarce, heterogeneous or biased, limiting the generalizability of the models. Additionally, the immunological systems being modelled are inherently complex, and oversimplification can lead to misleading predictions or overlooked interactions [21].

DT model validation and interpretability thus represent essential considerations when initiating those approaches. For DT‐generated predictions to be trusted and adopted by clinicians and regulatory bodies, they must be rigorously validated against experimental and clinical outcomes [60]. Also, the ‘black box’ nature of many AI‐based models makes it difficult to understand how specific predictions are made. This lack of transparency can not only hinder acceptance but also limit the ability to troubleshoot or refine models when discrepancies arise [61]. Ethical and regulatory dimensions also have to be considered. The use of patient data in DT‐driven research raises concerns about privacy, consent and data security. Ensuring compliance with ethical standards and regulatory requirements is thus essential to protect patients and maintain public trust [62]. As of today, the regulatory landscape for AI‐based medical tools is still evolving, with agencies such as the FDA and EMA developing new frameworks to assess the safety and efficacy of these technologies.

Another significant challenge is integrating AI and computational models into existing industrial and clinical workflows. Many healthcare settings lack the infrastructure, expertise, or resources to implement advanced computational modelling solutions. Resistance to change among human experts, the need for staff training, and the need to adapt legacy systems can slow adoption. To overcome these barriers, interdisciplinary collaboration and extensive training on the functionalities introduced by DT technology will be necessary, ensuring that advances translate into tangible benefits for patients.

In addition, community‐driven efforts to harmonise data and facilitate accessibility [e.g. Human Immune Atlas (60), Immunome (61), ImmPort (62)] should be reinforced and supported globally by policies and regulatory schemes that promote Open Science and FAIRness [58, 59].

Controlled vocabularies, ontologies, curated metadata schemes and standards should be developed and used where possible to maximise seamless integration across scales and sources. Data stewardship and sovereignty, especially for sensitive, patient‐specific data, should be taken into account and regulated on both national, European, and international levels.

## Future perspectives

The future of DT modelling to support better‐targeted drugs for complex IDs is poised for rapid adoption, driven by technological innovation and growing interdisciplinary collaboration. Expected technological advances will likely include the maturation of explainable AI (XAI) systems, which aim to make the outputs and decision‐making processes of AI‐powered models more transparent and interpretable for clinicians and researchers [60, 61]. This will not only foster greater trust and adoption in clinical settings but also facilitate regulatory approval.

On a technical ground, a current challenge is to generate DTs that integrate multi‐omics biological data with high‐resolution imaging data to create ever more sophisticated representations of the individual patient's disease. Additionally, real‐time data acquisition from wearable devices, implantable sensors, and advanced imaging technologies will provide continuous streams of high‐resolution patient data. Integration of all those sources will enable dynamic monitoring of immune responses and treatment effects, enabling timely adjustments to therapeutic strategies.

On a broader scale, population‐level data analysis will help identify emerging trends in immune‐related diseases, facilitate early intervention, and inform public health policy. Such capabilities may prove invaluable for responding to epidemics or for adapting immunisation strategies across diverse populations. The use of virtual patient models that simulate individual immune responses holds particular promise for tailoring treatments, predicting outcomes, and minimising adverse effects. As such, this individualised approach not only should improve patient outcomes but also has the potential to reduce healthcare costs and inform public health strategies on a larger scale.

However, realizing this vision will require addressing key research needs and fostering interdisciplinary collaborations. Ongoing operative efforts must focus on standardizing data collection, improving model generalizability, and ensuring ethical data use. Those points have been emphasized in the context of an international effort to build a DT of the human immune system, with particular focus on the physiological and pathological dimensions of inflammatory responses. Collaboration among clinicians, data scientists, engineers, ethicists, and regulatory bodies will be essential to bridge gaps between technological capabilities and real‐world applications [21, 63]. By uniting expertise across disciplines, DT technology should accelerate the translation of computational advances into safe, effective, and innovative therapies for a range of diseases, including IDs (68).

## Conclusions

By harnessing multi‐scale and personalised models of immune function and leveraging advanced computational techniques, DTs are currently accelerating the discovery and development of new therapies for IDs, thereby supporting the evolution towards precision medicine in many of those diseases. Broader adoption of DT technologies by medical experts across research, clinical and regulatory domains will require rigorous professional training and the updating of university curricula, both critical measures to ensuring that the next generation of health providers is knowledgeable about both the potential and limitations of AI‐assisted technologies.

Continued investment in interdisciplinary research, data standardisation, and ethical frameworks will be essential to ensure that these tools are robust, transparent and accessible. Eventually, by embracing these innovations, the biomedical and scientific communities can drive the next generation of breakthroughs in ID treatment, ultimately improving the lives of millions worldwide.

## Conflict of interest

The authors declare no conflict of interest.

## Author contributions

AN and PM conceived and designed the project; AN and PM conducted the literature review; AN and PM analysed and interpreted the data; AN created the Figure; AN and PM created the table; and AN and PM wrote the manuscript, edited and made manuscript revisions.

## References

[1] Buckley CD , Chernajovsky L , Chernajovsky Y , Modis LK , O'Neill LA , Brown D , Connor R , Coutts D , Waterman EA and Tak PP (2021) Immune‐mediated inflammation across disease boundaries: breaking down research silos. Nat Immunol 22, 1344–1348. 34675389 10.1038/s41590-021-01044-7

[2] Agrebi S and Larbi A (2020) Use of artificial intelligence in infectious diseases. In Artificial Intelligence in Precision Health ( Barh D , ed.), pp. 415–438. Elsevier, Amsterdam.

[3] Barturen G , Babaei S , Català‐Moll F , Martínez‐Bueno M , Makowska Z , Martorell‐Marugán J , Carmona‐Sáez P , Toro‐Domínguez D , Carnero‐Montoro E , Teruel M *et al*. (2021) Integrative analysis reveals a molecular stratification of systemic autoimmune diseases. Arthritis Rheumatol 73, 1073–1085. 33497037 10.1002/art.41610

[4] Moingeon P (2023) Artificial intelligence‐driven drug development against autoimmune diseases. Trends Pharmacol Sci 44, 411–424. 37268540 10.1016/j.tips.2023.04.005

[5] Sanz I and Lund F (2019) Complexity and heterogeneity ‐ the defining features of autoimmune disease. Curr Opin Immunol 61, iii–vi. 31813416 10.1016/j.coi.2019.11.006

[6] Shamji MH , Ollert M , Adcock IM , Bennett O , Favaro A , Sarama R , Riggioni C , Annesi‐Maesano I , Custovic A , Fontanella S *et al*. (2023) EAACI guidelines on environmental science in allergic diseases and asthma ‐ leveraging artificial intelligence and machine learning to develop a causality model in exposomics. Allergy 78, 1742–1757. 36740916 10.1111/all.15667

[7] Lee H‐S and Cleynen I (2019) Molecular profiling of inflammatory bowel disease: is it ready for use in clinical decision‐making? Cells 8, 535. 31167397 10.3390/cells8060535 PMC6627070

[8] Rider NL , Coffey M , Kurian A , Quinn J , Orange JS , Modell V and Modell F (2023) A validated artificial intelligence‐based pipeline for population‐wide primary immunodeficiency screening. J Allergy Clin Immunol 151, 272–279. 36243223 10.1016/j.jaci.2022.10.005

[9] Niarakis A , Ostaszewski M , Mazein A , Kuperstein I , Kutmon M , Gillespie ME , Funahashi A , Acencio ML , Hemedan A , Aichem M *et al*. (2023) Drug‐target identification in COVID‐19 disease mechanisms using computational systems biology approaches. Front Immunol 14, 1282859. 38414974 10.3389/fimmu.2023.1282859 PMC10897000

[10] Desvaux E , Hamon A , Hubert S , Boudjeniba C , Chassagnol B , Swindle J , Aussy A , Laigle L , Laplume J , Soret P *et al*. (2021) Network‐based repurposing identifies anti‐alarmins as drug candidates to control severe lung inflammation in COVID‐19. PLoS One 16, e0254374. 34293006 10.1371/journal.pone.0254374 PMC8297899

[11] Nigrovic PA , Lee PY and Hoffman HM (2020) Monogenic autoinflammatory disorders: conceptual overview, phenotype, and clinical approach. J Allergy Clin Immunol 146, 925–937. 33160483 10.1016/j.jaci.2020.08.017 PMC7727443

[12] An J , Marwaha A and Laxer RM (2024) Autoinflammatory diseases: a review. J Rheumatol 51, 848–861. 38879186 10.3899/jrheum.2023-1209

[13] Guthridge JM , Wagner CA and James JA (2022) The promise of precision medicine in rheumatology. Nat Med 28, 1363–1371. 35788174 10.1038/s41591-022-01880-6 PMC9513842

[14] Moingeon P (2024) Harnessing the power of AI‐based models to accelerate drug discovery against immune diseases. Expert Rev Clin Immunol 20, 1135–1138. 38932714 10.1080/1744666X.2024.2373915

[15] Obermeyer Z and Emanuel EJ (2016) Predicting the future – big data, machine learning, and clinical medicine. N Engl J Med 375, 1216–1219. 27682033 10.1056/NEJMp1606181 PMC5070532

[16] LeCun Y , Bengio Y and Hinton G (2015) Deep learning. Nature 521, 436–444. 26017442 10.1038/nature14539

[17] Moingeon P , Kuenemann M and Guedj M (2022) Artificial intelligence‐enhanced drug design and development: toward a computational precision medicine. Drug Discov Today 27, 215–222. 34555509 10.1016/j.drudis.2021.09.006

[18] Vallée A (2023) Digital twin for healthcare systems. Front Digit Health 5, 1253050. 37744683 10.3389/fdgth.2023.1253050 PMC10513171

[19] Laubenbacher R , Niarakis A , Helikar T , An G , Shapiro B , Malik‐Sheriff RS , Sego TJ , Knapp A , Macklin P and Glazier JA (2022) Building digital twins of the human immune system: toward a roadmap. NPJ Digit Med 5, 64. 35595830 10.1038/s41746-022-00610-z PMC9122990

[20] Puniya BL (2025) Artificial‐intelligence‐driven innovations in mechanistic computational modeling and digital twins for biomedical applications. J Mol Biol 437, 169181. 40316010 10.1016/j.jmb.2025.169181

[21] Niarakis A , Laubenbacher R , An G , Ilan Y , Fisher J , Flobak Å , Reiche K , Rodríguez Martínez M , Geris L , Ladeira L *et al*. (2024) Immune digital twins for complex human pathologies: applications, limitations, and challenges. NPJ Syst Biol Appl 10, 141. 39616158 10.1038/s41540-024-00450-5 PMC11608242

[22] Alsaedi S , Gao X and Gojobori T (2025) Beyond digital twins: the role of foundation models in enhancing the interpretability of multiomics modalities in precision medicine. FEBS Open Bio 15, 1192–1208. 10.1002/2211-5463.70003 PMC12319712 39995150

[23] Zerrouk N , Augé F and Niarakis A (2024) Building a modular and multi‐cellular virtual twin of the synovial joint in rheumatoid arthritis. NPJ Digit Med 7, 379. 39719524 10.1038/s41746-024-01396-y PMC11668869

[24] Johnson JAI , Bergman DR , Rocha HL , Zhou DL , Cramer E , Mclean IC , Dance YW , Booth M , Nicholas Z , Lopez‐Vidal T *et al*. (2025) Human interpretable grammar encodes multicellular systems biology models to democratize virtual cell laboratories. Cell 188, 4711–4733.e37 40713951 10.1016/j.cell.2025.06.048 PMC13012569

[25] An G (2008) Introduction of an agent‐based multi‐scale modular architecture for dynamic knowledge representation of acute inflammation. Theor Biol Med Model 5, 11. 18505587 10.1186/1742-4682-5-11 PMC2442588

[26] Germain RN , Meier‐Schellersheim M , Nita‐Lazar A and Fraser IDC (2011) Systems biology in immunology: a computational modeling perspective. Annu Rev Immunol 29, 527–585. 21219182 10.1146/annurev-immunol-030409-101317 PMC3164774

[27] Hall BA and Niarakis A (2021) Data integration in logic‐based models of biological mechanisms. Curr Opin Syst Biol 28, 100386.

[28] Mason DM , Friedensohn S , Weber CR , Jordi C , Wagner B , Meng SM , Ehling RA , Bonati L , Dahinden J , Gainza P *et al*. (2021) Optimization of therapeutic antibodies by predicting antigen specificity from antibody sequence via deep learning. Nat Biomed Eng 5, 600–612. 33859386 10.1038/s41551-021-00699-9

[29] Moingeon P , Chenel M , Rousseau C , Voisin E and Guedj M (2023) Virtual patients, digital twins and causal disease models: paving the ground for in silico clinical trials. Drug Discov Today 28, 103605. 37146963 10.1016/j.drudis.2023.103605

[30] Shannon P , Markiel A , Ozier O , Baliga NS , Wang JT , Ramage D , Amin N , Schwikowski B and Ideker T (2003) Cytoscape: a software environment for integrated models of biomolecular interaction networks. Genome Res 13, 2498–2504. 14597658 10.1101/gr.1239303 PMC403769

[31] Zerrouk N , Aghakhani S , Singh V , Augé F and Niarakis A (2022) A mechanistic cellular atlas of the rheumatic joint. Front Syst Biol 2, 925791.

[32] Zerrouk N , Miagoux Q , Dispot A , Elati M and Niarakis A (2020) Identification of putative master regulators in rheumatoid arthritis synovial fibroblasts using gene expression data and network inference. Sci Rep 10, 16236. 33004899 10.1038/s41598-020-73147-4 PMC7529794

[33] Miagoux Q , Singh V , de Mézquita D , Chaudru V , Elati M , Petit‐Teixeira E and Niarakis A (2021) Inference of an integrative, executable network for rheumatoid arthritis combining data‐driven machine learning approaches and a state‐of‐the‐art mechanistic disease map. J Pers Med 11, 785. 34442429 10.3390/jpm11080785 PMC8400381

[34] Müller‐Dott S , Tsirvouli E , Vazquez M , Ramirez Flores RO , Badia‐I‐Mompel P , Fallegger R , Türei D , Lægreid A and Saez‐Rodriguez J (2023) Expanding the coverage of regulons from high‐confidence prior knowledge for accurate estimation of transcription factor activities. Nucleic Acids Res 51, 10934–10949. 37843125 10.1093/nar/gkad841 PMC10639077

[35] Zerrouk N , Alcraft R , Hall BA , Augé F and Niarakis A (2024) Large‐scale computational modelling of the M1 and M2 synovial macrophages in rheumatoid arthritis. NPJ Syst Biol Appl 10, 10. 38272919 10.1038/s41540-024-00337-5 PMC10811231

[36] Tsirvouli E , Ashcroft F , Johansen B and Kuiper M (2021) Logical and experimental modeling of cytokine and eicosanoid signaling in psoriatic keratinocytes. iScience 24, 103451. 34877506 10.1016/j.isci.2021.103451 PMC8633970

[37] Niarakis A and Helikar T (2021) A practical guide to mechanistic systems modeling in biology using a logic‐based approach. Brief Bioinform 22, bbaa236. 33064138 10.1093/bib/bbaa236 PMC8293813

[38] Abramson J , Adler J , Dunger J , Evans R , Green T , Pritzel A , Ronneberger O , Willmore L , Ballard AJ , Bambrick J *et al*. (2024) Accurate structure prediction of biomolecular interactions with AlphaFold 3. Nature 630, 493–500. 38718835 10.1038/s41586-024-07487-w PMC11168924

[39] Schardt JS , Jhajj HS , O'Meara RL , Lwo TS , Smith MD and Tessier PM (2022) Agonist antibody discovery: experimental, computational, and rational engineering approaches. Drug Discov Today 27, 31–48. 34571277 10.1016/j.drudis.2021.09.008 PMC8714685

[40] Myers RC , Augustin F , Huard J and Friedrich CM (2023) Using machine learning surrogate modeling for faster QSP VP cohort generation. CPT Pharmacometrics Syst Pharmacol 12, 1047–1059. 37328956 10.1002/psp4.12999 PMC10431055

[41] Hurez V , Gauderat G , Soret P , Myers R , Dasika K , Sheehan R , Friedrich C , Reed M , Laigle L , Riquelme MA *et al*. (2025) Virtual patients inspired by multiomics predict the efficacy of an anti‐IFNα mAb in cutaneous lupus. iScience 28, 111754. 39925417 10.1016/j.isci.2025.111754 PMC11804754

[42] Singh V , Naldi A , Soliman S and Niarakis A (2023) A large‐scale Boolean model of the rheumatoid arthritis fibroblast‐like synoviocytes predicts drug synergies in the arthritic joint. NPJ Syst Biol Appl 9, 33. 37454172 10.1038/s41540-023-00294-5 PMC10349856

[43] Soret P , Le Dantec C , Desvaux E , Foulquier N , Chassagnol B , Hubert S , Jamin C , Barturen G , Desachy G , Devauchelle‐Pensec V *et al*. (2021) A new molecular classification to drive precision treatment strategies in primary Sjögren's syndrome. Nat Commun 12, 3523. 34112769 10.1038/s41467-021-23472-7 PMC8192578

[44] Mu C , Zhao Q , Zhao Q , Yang L , Pang X , Liu T , Li X , Wang B , Fung S‐Y and Cao H (2023) Multi‐omics in Crohn's disease: new insights from inside. Comput Struct Biotechnol J 21, 3054–3072. 37273853 10.1016/j.csbj.2023.05.010 PMC10238466

[45] Guedj M , Swindle J , Hamon A , Hubert S , Desvaux E , Laplume J , Xuereb L , Lefebvre C , Haudry Y , Gabarroca C *et al*. (2022) Industrializing AI‐powered drug discovery: lessons learned from the patrimony computing platform. Expert Opin Drug Discov 17, 815–824. 35786124 10.1080/17460441.2022.2095368

[46] Desvaux E , Aussy A , Hubert S , Keime‐Guibert F , Blesius A , Soret P , Guedj M , Pers J‐O , Laigle L and Moingeon P (2022) Model‐based computational precision medicine to develop combination therapies for autoimmune diseases. Expert Rev Clin Immunol 18, 47–56. 34842494 10.1080/1744666X.2022.2012452

[47] Laigle L , Chadli L and Moingeon P (2023) Biomarker‐driven development of new therapies for autoimmune diseases: current status and future promises. Expert Rev Clin Immunol 19, 305–314. 36680799 10.1080/1744666X.2023.2172404

[48] Voigt I , Inojosa H , Dillenseger A , Haase R , Akgün K and Ziemssen T (2021) Digital twins for multiple sclerosis. Front Immunol 12, 669811. 34012452 10.3389/fimmu.2021.669811 PMC8128142

[49] Scott J , Grant‐Jacob JA , Praeger M , Coltart G , Sutton J , Zervas MN , Niranjan M , Eason RW , Healy E and Mills B (2025) Modifying the severity and appearance of psoriasis using deep learning to simulate anticipated improvements during treatment. Sci Rep 15, 7412. 40032873 10.1038/s41598-025-91238-y PMC11876654

[50] Reiche K , Weirauch U , Kreuz M , Fischer L , Gras L , Neumuth T , Platzbecker U , Köhl U , Demlova R , Kremer A *et al*. (2025) Virtual twins for personalised CAR T‐cell therapy in myeloma. Lancet Haematol 12, e490–e491. 40610172 10.1016/S2352-3026(25)00170-X

[51] Bouget V , Duquesne J , Hassler S , Cournède P‐H , Fautrel B , Guillemin F , Pallardy M , Broët P , Mariette X and Bitoun S (2022) Machine learning predicts response to TNF inhibitors in rheumatoid arthritis: results on the ESPOIR and ABIRISK cohorts. RMD Open 8, e002442. 35999028 10.1136/rmdopen-2022-002442 PMC9403109

[52] Susilo ME , Li C‐C , Gadkar K , Hernandez G , Huw L‐Y , Jin JY , Yin S , Wei MC , Ramanujan S and Hosseini I (2023) Systems‐based digital twins to help characterize clinical dose‐response and propose predictive biomarkers in a phase I study of bispecific antibody, mosunetuzumab, in NHL. Clin Transl Sci 16, 1134–1148. 36908269 10.1111/cts.13501 PMC10339700

[53] Shen S , Qi W , Liu X , Zeng J , Li S , Zhu X , Dong C , Wang B , Shi Y , Yao J *et al*. (2025) From virtual to reality: innovative practices of digital twins in tumor therapy. J Transl Med 23, 348. 40108714 10.1186/s12967-025-06371-z PMC11921680

[54] Saria S (2018) Individualized sepsis treatment using reinforcement learning. Nat Med 24, 1641–1642. 30397359 10.1038/s41591-018-0253-x

[55] An G and Cockrell C (2024) A design specification for Critical Illness Digital Twins (CIDTs) to cure sepsis: responding to the National Academies of Sciences, Engineering and Medicine Report “Foundational Research Gaps and Future Directions for Digital Twins”. *arXiv*. 39088664

[56] Jayatunga M , Ayers M , Bruens L , Jayanth D and Meier C (2024) How successful are AI‐discovered drugs in clinical trials? A first analysis and emerging lessons. Drug Discov Today 29, 104009. 38692505 10.1016/j.drudis.2024.104009

[57] Pulendran B (2025) The creation game: of AI and human creativity. Nat Immunol 26, 1–2. 39747428 10.1038/s41590-024-02026-1

[58] Sauro HM , Agmon E , Blinov ML , Gennari JH , Hellerstein J , Heydarabadipour A , Hunter P , Jardine BE , May E , Nickerson DP *et al*. (2025) From FAIR to CURE: guidelines for computational models of biological systems. *arXiv*. 10.1038/s41540-026-00651-0 PMC13230930 41888157

[59] Niarakis A , Waltemath D , Glazier J , Schreiber F , Keating SM , Nickerson D , Chaouiya C , Siegel A , Noël V , Hermjakob H *et al*. (2022) Addressing barriers in comprehensiveness, accessibility, reusability, interoperability and reproducibility of computational models in systems biology. Brief Bioinform 23, bbac212. 35671510 10.1093/bib/bbac212 PMC9294410

[60] Tonekaboni S , Joshi S , McCradden MD and Goldenberg A (2019) What clinicians want: contextualizing explainable machine learning for clinical end use. *arXiv*.

[61] Holzinger A , Langs G , Denk H , Zatloukal K and Müller H (2019) Causability and explainability of artificial intelligence in medicine. Wiley Interdiscip Rev Data Min Knowl Discov 9, e1312. 32089788 10.1002/widm.1312 PMC7017860

[62] Meijer C , Uh H‐W and El Bouhaddani S (2023) Digital twins in healthcare: methodological challenges and opportunities. J Pers Med 13, 1522. 37888133 10.3390/jpm13101522 PMC10608065

[63] Niarakis A , An G , Ladeira L , Hiroi NF , Papadopoulou A , Crawley FP , Nikaein N , Calzone L , Tsirvouli E , Balci H *et al*. (2025) Building immune digital twins: an international and transdisciplinary community effort. ImmunoInformatics 20, 100060.

[64] Cockrell C and An G (2021) Utilizing the heterogeneity of clinical data for model refinement and rule discovery through the application of genetic algorithms to calibrate a high‐dimensional agent‐based model of systemic inflammation. Front Physiol 12, 662845. 34093225 10.3389/fphys.2021.662845 PMC8172123

[65] Aghakhani S , Soliman S and Niarakis A (2022) Metabolic reprogramming in rheumatoid arthritis synovial fibroblasts: a hybrid modeling approach. PLoS Comput Biol 18, e1010408. 36508473 10.1371/journal.pcbi.1010408 PMC9779668

[66] An G and Cockrell C (2024) A design specification for Critical Illness Digital Twins to cure sepsis: responding to the National Academies of Sciences, Engineering and Medicine Report: Foundational Research Gaps and Future Directions for Digital Twins. *arXiv*. 39088664

